# Editorial: Approaches to the management of weight regain after bariatric surgery

**DOI:** 10.3389/fendo.2023.1267014

**Published:** 2023-08-14

**Authors:** Meera Shah, Jaime P. Almandoz, Alpana P. Shukla

**Affiliations:** ^1^ Division of Endocrinology, Diabetes, & Metabolism, Mayo Clinic College of Medicine, Rochester, MN, United States; ^2^ Department of Internal Medicine, Division of Endocrinology, UT Southwestern Medical Center, Dallas, United States; ^3^ Comprehensive Weight Control Center, Division of Endocrinology, Diabetes, & Metabolism, Weill Cornell Medicine, New York, NY, United States

**Keywords:** weight regain after bariatric surgery, antiobesity medications, endoscopic therapy, weight stigma, eating behaviors

Bariatric surgery is one of the most effective interventions for treating obesity and weight-related complications. Since the early days of bariatric surgery, several innovations in surgical technique and periprocedural management have improved overall outcomes and reduced the risk of short and long-term complications. Bariatric surgery continues to be the treatment of choice for many patients with medically complicated obesity in whom lifestyle interventions, with or without anti-obesity medications, have been insufficient.

Weight regain is a recognized complication of bariatric surgery that affects about 20 to 25% of patients and is more prevalent after the second postoperative year (1). Weight regain can be associated with a recurrence of weight-related complications (2). Beyond the impact of weight gain on cardiometabolic, biomechanical, and mental health complications of obesity, it can also have a significant negative impact on quality of life (3). In this Research Topic, leading authors in the field discuss a multi-modal approach to the management of post-bariatric weight regain, which occurs through complex mechanisms that can be challenging to treat.

‘Behavioral Interventions to Attenuate Driven Overeating and Weight Regain after Bariatric Surgery’ focuses on identifying and classifying eating behaviors after bariatric surgery that lead to over-consumption of calories (Ames et al.). Recognizing problematic eating behaviors and the complexity of eating behaviors offers an opportunity to tailor a behavioral treatment strategy that is effective for the patient.

Anti-obesity medications are effective therapies for treating post-bariatric weight regain. In ‘Pharmacologic Management of Weight Regain Following Bariatric Surgery‘, the authors discuss the evidence supporting the use of anti-obesity medications for weight regain in patients who have undergone bariatric surgery (Lucas et al.) This is an area of immense growth, and as more patients undergo bariatric surgery and the number of available anti-obesity medications increases, we will learn more about the efficacy, safety, and scope of these medications for this understudied patient population.

‘Endoscopic Management of Weight Recurrence Following Bariatric Surgery’ highlights the role of minimally invasive, endoscopic options for managing weight regain in select patients (Abboud et al.). This review highlights short and long-term weight loss outcomes and safety data associated with these emerging procedures. As with other treatment modalities, endoscopic therapies show superior outcomes when paired with a multidisciplinary approach to weight regain, which reinforces the importance of a comprehensive treatment strategy for patients with weight regain after bariatric surgery.

A patient’s lived experience with obesity, including their interactions with the healthcare system, influences how and whether they choose to seek treatment for weight regain following bariatric surgery. This is complicated by the high level of attrition to follow-up after bariatric surgery, which may be impacted by experienced weight stigma and bias. In ‘The Role of Weight Stigma in Weight Regain in Bariatric Surgery’ the authors discuss the critical role of societal messaging, as well as messaging within the medical community towards postsurgical patients that can affect adherence and follow-up (Himmelstein et al.). Understanding the contributors to weight bias internalization can help improve the patient-provider relationship and may reduce delays in patients with weight regain seeking care.

Bariatric surgery is a safe, effective, and durable therapy for obesity and its complications. Beyond improving health, bariatric surgery also increases quality of life and life expectancy. However, obesity is a chronic disease and bariatric surgery is not curative. Significant weight regain is a reality for a small proportion of patients who undergo bariatric surgery and may be challenging to treat. The optimal management of post-bariatric weight regain acknowledges the complexity of the contributing physiological, behavioral, and anatomical factors, and that a multidisciplinary team approach can increase the likelihood of optimizing weight, health outcomes, and quality of life.

## Author contributions

AS: Writing – review & editing. MS: Writing – original draft. JA: Writing – review & editing.
